# Contribution of volatile organic compound fluxes to the ecosystem carbon budget of a poplar short‐rotation plantation

**DOI:** 10.1111/gcbb.12506

**Published:** 2018-03-25

**Authors:** Miguel Portillo‐Estrada, Terenzio Zenone, Nicola Arriga, Reinhart Ceulemans

**Affiliations:** ^1^ Centre of Excellence PLECO Department of Biology University of Antwerp Universiteitsplein 1 Wilrijk B‐2610 Belgium

**Keywords:** eddy covariance, gross primary production, isoprene emission, methanol emission, net ecosystem exchange, proton‐transfer‐reaction, mass spectrometry

## Abstract

Biogenic volatile organic compounds (BVOCs) are major precursors of both ozone and secondary organic aerosols (SOA) in the troposphere and represent a non‐negligible portion of the carbon fixed by primary producers, but long‐term ecosystem‐scale measurements of their exchanges with the atmosphere are lacking. In this study, the fluxes of 46 ions corresponding to 36 BVOCs were continuously monitored along with the exchanges of mass (carbon dioxide and water vapor) and energy (sensible and latent heat) for an entire year in a poplar (*Populus*) short‐rotation crop (SRC), using the eddy covariance methodology. BVOC emissions mainly consisted of isoprene, acetic acid, and methanol. Total net BVOC emissions were 19.20 kg C ha^−1^ yr^−1^, which represented 0.63% of the net ecosystem exchange (NEE), resulting from −23.59 Mg C ha^−1^ yr^−1^ fixed as CO_2_ and 20.55 Mg C ha^−1^ yr^−1^ respired as CO_2_ from the ecosystem. Isoprene emissions represented 0.293% of NEE, being emitted at a ratio of 1 : 1709 mol isoprene per mol of CO_2_ fixed. Based on annual ecosystem‐scale measurements, this study quantified for the first time that BVOC carbon emissions were lower than previously estimated in other studies (0.5–2% of NEE) on poplar trees. Furthermore, the seasonal and diurnal emission patterns of isoprene, methanol, and other BVOCs provided a better interpretation of the relationships with ecosystem CO_2_ and water vapor fluxes, with air temperature, vapor pressure deficit, and photosynthetic photon flux density.

## Introduction

Plants exchange a wide array of biogenic volatile organic compounds (BVOCs) with the atmosphere (Kesselmeier & Staudt, 1999). On a global scale, these BVOC emissions were estimated to be an order of magnitude larger than those emitted from anthropogenic sources. Annually 1150 Tg of carbon assimilated by primary producers is emitted back to the atmosphere as BVOCs, and in particular, 500 Tg of carbon is emitted as isoprene (Guenther *et al*., 1995). BVOCs play a role in several ecological processes and are major precursors of both ozone and secondary organic aerosols (SOA) in the troposphere (Ashworth *et al*., 2012). Considering the ecological and atmospheric importance, the exchange of BVOCs needs to be adequately measured and represented to accurately model and understand the coupled biosphere–atmosphere interactions with photosynthetic activities and CO_2_ exchanges (Seco *et al*., 2015).

Photosynthesis and isoprene emission are particularly closely linked as photosynthetic carbon is the main source of isoprene (Sharkey & Yeh, 2001). Therefore, the high photosynthetic capacity (10–20 μmol m^−2^ s^−1^) that characterizes the *Populus* genus might explain its high isoprene emission rates (20–45 nmol m^−2^ s^−1^) in leaf cuvettes at optimal temperature and under saturating light (Guidolotti *et al*., 2011; Rasulov *et al*., 2015). Studies have shown a temporal lag between isoprene emission and the onset of photosynthesis (Kuzma & Fall, 1993; Sharkey & Loreto, 1993; Monson *et al*., 1995). Leaf phenology plays an important role in determining the level of isoprene emissions (Brilli *et al*., 2016; Portillo‐Estrada *et al*., 2017). Isoprene emission rates differ among different species of the same genus, or among genotypes of the same species, as already documented in *Populus* (Calfapietra *et al*., 2008). The release of carbon into the atmosphere in the form of BVOCs from vegetation is estimated around 0.1–2% of the carbon assimilated by photosynthesis (Sharkey & Yeh, 2001) while these values may reach 1–3% in poplar species (Goldstein *et al*., 1998).

Plantations of short‐rotation poplar crops (SRC) dedicated for the production of bioenergy are characterized by a considerable amount of BVOCs emitted mainly in the form of isoprene (Zenone *et al*., 2016) and methanol (Brilli *et al*., 2016). Previous studies (Brilli *et al*., 2014, 2016) have shown that SRC is a net, albeit very small (~1 g m^−2^ per growing season) BVOC carbon source to the atmosphere. Long‐term ecosystem‐scale studies of BVOC fluxes are scarce, although there have been some short‐term studies (e.g., Brilli *et al*. (2014), Misztal *et al*. (2011), Schallhart *et al*. (2016)). To our knowledge, this study is the first one that continuously monitored and quantified the fluxes of many BVOC species together with CO_2_ fluxes during a whole year.

This study aimed (i) to quantify the net annual carbon emitted as BVOCs in a poplar SRC, while at the same time evaluating the individual contributions of each BVOC species, (ii) to calculate the year‐based ratio of carbon emitted as BVOCs to the net ecosystem exchange of carbon, and (iii) to investigate the relationships of the fluxes of isoprene and other major BVOCs with CO_2_ fluxes and meteorological variables.

## Materials and methods

### Study site

The study was conducted at an existing poplar (*Populus* spp.) plantation managed as a SRC located in Lochristi, Belgium (51°6′44″N, 3°51′2″E), at an elevation of 6 m.a.s.l. The 11‐ha plantation contained monoclonal rows of 12 different interspecific genotypes of *Populus deltoides*,* P*. *maximowiczii*,* P*. *nigra*, and *P*. *trichocarpa*, with a stand density of 8000 trees ha^−1^. In the year 2015, the poplar plantation was in its second year of the third rotation. More details of the plantation, site characteristics, and genotypic features have been previously published (Broeckx *et al*., 2012; Verlinden *et al*., 2015). The long‐term average annual and growing season temperature at the site are 9.5 and 13.72 °C, respectively. Average annual and growing season precipitation are 726 mm and 433 mm, respectively (Broeckx *et al*., 2012). For this study, the growing season was defined as the period of the year when weekly averaged net ecosystem exchange (NEE) was negative, that is, net ecosystem carbon uptake. All measurements of this study were collected between January 1, 2015 and December 31, 2015.

Meteorological data were measured on an extendable meteorological mast at the site and recorded at half‐hourly time steps. Air temperature (°C) and air relative humidity (%) were measured by Vaisala probes (model HMP45C; Vaisala, Helsinki, Finland) and used to calculate vapor pressure deficit (*VPD*; kPa) (Allen *et al*., 2005). Photosynthetic photon flux density (PPFD) was measured by a photodiode‐based radiometer active in the PPFD wavelength range, that is, 400 nm to 700 nm (model LI‐190R, LI‐COR Biosciences Inc., Lincoln, NE, USA).

### Eddy covariance measurements of BVOCs and CO_2_

A proton‐transfer‐reaction time‐of‐flight mass spectrometer (PTR‐TOF‐MS Ionicon, Innsbruck, Austria) to measure the volume mixing ratios of BVOCs at a frequency suitable for the eddy covariance technique was co‐located with a sonic anemometer (HS‐50, Gill Instruments Ltd, Lymington, Hampshire, UK) to measure the tri‐dimensional direction of wind speed. The data of both instruments were recorded by two independent logging devices, synchronized to each other with a dedicated software (NTP, Network Time Protocol, University of Delaware, DE, USA) and to an external clock through the Internet with an accuracy of <20 ms. The volume mixing ratios of CO_2_ and H_2_O used to estimate NEE and latent heat fluxes (LE), respectively, were measured using a fast‐response CO_2_/H_2_O infrared analyser (LI‐7200, LI‐COR Biosciences Inc., Lincoln, NE, USA) at a 10 Hz rate. A continuous air stream was drawn through a 25‐m PTFE (polytetrafluoroethylene) tube (6 mm inner diameter) by a pump at a constant flow rate of ≈20 L min^−1^. The pipeline was heated to ≈40 °C to prevent condensation of water within the pipe. The flow was diverted to the PTR‐TOF‐MS through a 1/32 inch ID (1/16 inch OD) capillary heated to 60 °C at a flow rate of 74.4 μmol s^−1^. The sample air continuously entered the PTR‐TOF‐MS and met a rich H_3_O^+^ mixture in a chamber where the proton‐transfer‐reaction takes place. Unavoidably the H_3_O^+^ mixture contained small amounts of parasitic reagent ions as NO^+^, O_2_^+^, and water cluster H_2_O‐H_3_O^+^. Protonated molecules were driven through a drift tube operated at 600 V of electric potential, 2.3 mbar of pressure, and 60 °C of temperature, resulting in a field density ratio (E/N) of ≈130 Td. The ions exiting the drift tube were pulsed every 32000 ns to the time‐of‐flight region and separated by their *m*/*z* ratio. A total of 3125 spectra ranging from *m*/*z* of 0–316 were averaged every 100 ms, resulting in 10 Hz data (32000 ns spectrum^−1^ × 3125 spectra = 0.1 s). The data recording was automated as shown in Fig. S1.

The mass range of the spectra was automatically calibrated during the measurement process by identifying the peak center of hydronium isotope (H_2_^18^O‐H^+^) as *m*/*z = *21.0221 and the peak center of diiodobenzene fragment (C_6_H_4_I‐H^+^) as *m*/*z = *203.9431, which was continuously permeated to the air inlet. The PTR‐TOF‐MS had higher values of ion transmission (fraction of ions detected to the initial amount of ions entered) for heavier masses than for lighter masses. Therefore, every month, a mixture of ten pure species containing acetaldehyde, methanol, isoprene, acetone, methyl vinyl ketone, benzene, toluene, t‐2‐hexen‐1‐al, c‐3‐hexen‐1‐ol, and α‐pinene (masses ranging from m^+^/z 33 to 137) was used to determine the transmission values at certain molecular masses. The transmission curve was later used to inter‐ and extrapolate the transmission values for each BVOC and to calculate the original amount of molecules entering the PTR‐TOF‐MS.

Each peak of the spectrum was fitted to a Gaussian function and its signal integrated. The Gaussian function was also used to discriminate the signal of adjacent peaks in multipeak systems (Portillo‐Estrada *et al*., 2015b) (example shown in Fig. S2). The 10 Hz spectra were individually processed with PTR‐MS Viewer v3.2 (Ionicon, Innsbruck, Austria) to extract the peak area of each ion. The concentration of each compound was calculated based on the peak area of the protonated parent ion, the concentration of H_3_O^+^ of the current spectrum (measured by hydronium isotope), the ion transmission factor at a given molecular mass explained above, and the reaction rate coefficient of each BVOC (explained further below), expressed in ppb at a given time. This laborious step (~4.3 million peaks per day of data) was facilitated by the ‘automation tool’ of the software. The files with volume mixing ratio values were then exported to .txt files and merged half‐hourly. A total of 46 ion peaks corresponding to 36 BVOCs and their major fragments were selected (see Table S1) to characterize the carbon fluxes as BVOCs.

The coefficients of reaction between each BVOC and H_3_O^+^ were either calculated via a calibration with the above‐mentioned gas standard mixture, retrieved from the Supplementary Information of Cappellin *et al*. (2012), or otherwise they were assumed to be 2 × 10^−9^ cm^3^ s^−1^.

### Calculation of CO_2_ and BVOC fluxes

Fluxes were estimated by applying the following processing options among the available solutions (Rebmann *et al*., 2012). Turbulent fluctuations and statistical moments, that is, variance and covariance, were calculated using the linear detrending method. Time lags between wind speed components and CO_2_ and H_2_O mixing ratios were estimated using the covariance maximization method. The coordinate reference system was rotated to align it with the mean wind direction applying the 2D rotations. The spectral corrections for high‐frequency attenuation were executed using the method proposed by Ibrom *et al*. (2007) to correct for signal attenuation mainly due to path averaging and scalar attenuation and the method of Horst & Lenschow (2009) to account for the spatial displacement of the sensors. All this processing was executed with the eddypro software version 5.1.1, freely available from LI‐COR Biosciences Inc., Lincoln, NE, USA (https://www.licor.com/env/products/eddy_covariance/compute.html#eddypro).

The anemometer and PTR‐TOF‐MS time series were merged and synchronized with a dedicated MATLAB code and then processed with eddypro applying a processing scheme similar to CO_2_ and H_2_O fluxes. The time lag was estimated only for isoprene and then applied to all other BVOC species, assuming that all BVOCs were subject to the same physical turbulent transport across the sampling line (Fares *et al*., 2012).

### Data series gap‐filling and partitioning method

Quality controls on half‐hourly fluxes were applied according to the methodology reported in Mauder & Foken (2015): The flag attained the value ‘0’ for the best quality fluxes, ‘1’ for fluxes suitable for general analysis such as annual budgets, and ‘2’ for fluxes that were discarded from the results dataset. Missing values of BVOC fluxes were gap filled using the mean diurnal variation technique as proposed by Bamberger *et al*. (2014). The missing half‐hourly values were replaced by the corresponding half‐hourly values of the average diurnal cycle calculated within a time window of ±8 days around the missing values. Ecosystem respiration (R_eco_) and gross primary production (GPP) half‐hourly values were estimated using nighttime CO_2_ flux data applying the partitioning procedures defined by Reichstein *et al*. (2005). GPP represents the total uptake of CO_2_ by the ecosystem. Missing or noncorrect values of measured NEE were gap filled to obtain complete yearly time series of NEE, R_eco,_ and GPP using the freely available routine of the Max Planck Institute for Biogeochemistry (https://www.bgc-jena.mpg.de/bgi/index.php/Services/REddyProcWebRPackage).

## Results

### Partitioning of ecosystem carbon fluxes

The amount of net C emitted as BVOCs was 19.20 kg C ha^−1^ yr^−1^, which represented 0.63% of NEE. Ecosystem GPP was −23.59 Mg C ha^−1^ yr^−1^, R_eco_ was 20.55 Mg C ha^−1^ yr^−1^, and NEE (the difference between GPP and R_eco_) was −3.04 Mg C ha^−1^ yr^−1^ for the year 2015. The growing season started after DOY 80 (March 21), when GPP became larger than R_eco_. According to the aforementioned definition, the growing season ended at DOY 270 (September 27), after which R_eco_ became larger than GPP until the end of the year 2015.

The length of the measurement period provides high confidence when averaging the carbon allocated to BVOC emissions. If this experiment would have lasted only the growing season, where the cumulative NEE was −5.82 Mg C ha^−1^ and BVOC carbon emission was 19.21 kg C ha^−1^, BVOCs would represent only 0.33% of NEE, which is half the year‐long average. Furthermore, the weekly result of BVOC allocated carbon varied considerably during the growing season, from 0.09 to 1.14% of NEE, being the low values due to low BVOC emissions at the beginning and the end of the growing season.

### Net annual emissions of BVOCs

**Figure 1:**
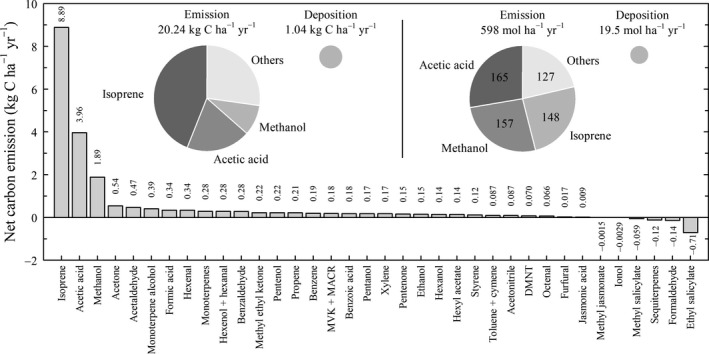
Annual net emissions of the most relevant biogenic volatile organic compounds (BVOCs) expressed as mass of carbon atoms. The insets are pie charts divided into sections proportional in size to the contribution of the main BVOCs to the annual fluxes expressed as (left) mass of carbon atoms and (right) amount of molecules. MVK, methyl vinyl ketone; MACR, methacrolein; and DMNT, dimethyl nonatriene. See Table S1 for more information on the BVOCs.

Annual net isoprene emission represented 0.293% of assimilated carbon (in terms of mass), and stoichiometrically, one molecule of isoprene was emitted for every 1709 molecules of CO_2_ fixed (i.e., 148.1 mol isoprene ha^−1^ yr^−1^ divided by 253167 mol CO_2_ ha^−1^ yr^−1^). Total BVOC emissions were mainly dominated by isoprene, acetic acid, and methanol, which together made up 73% of the carbon emitted and a total of 79% of all molecules emitted (Fig. 1). The net annual emission levels of isoprene and methanol were similar in terms of molecules, but once transformed to mass of carbon, isoprene contributed five times more than methanol to the carbon emissions, due to the five atoms of carbon contained in isoprene molecules. The individual contribution of other volatiles was minor in terms of carbon as well as in amount of molecules. Few compounds (e.g., formaldehyde and ethyl salicylate) resulted in a negative net flux (Fig. 1).

### Seasonal variations in the emission of major BVOCs

**Figure 2:**
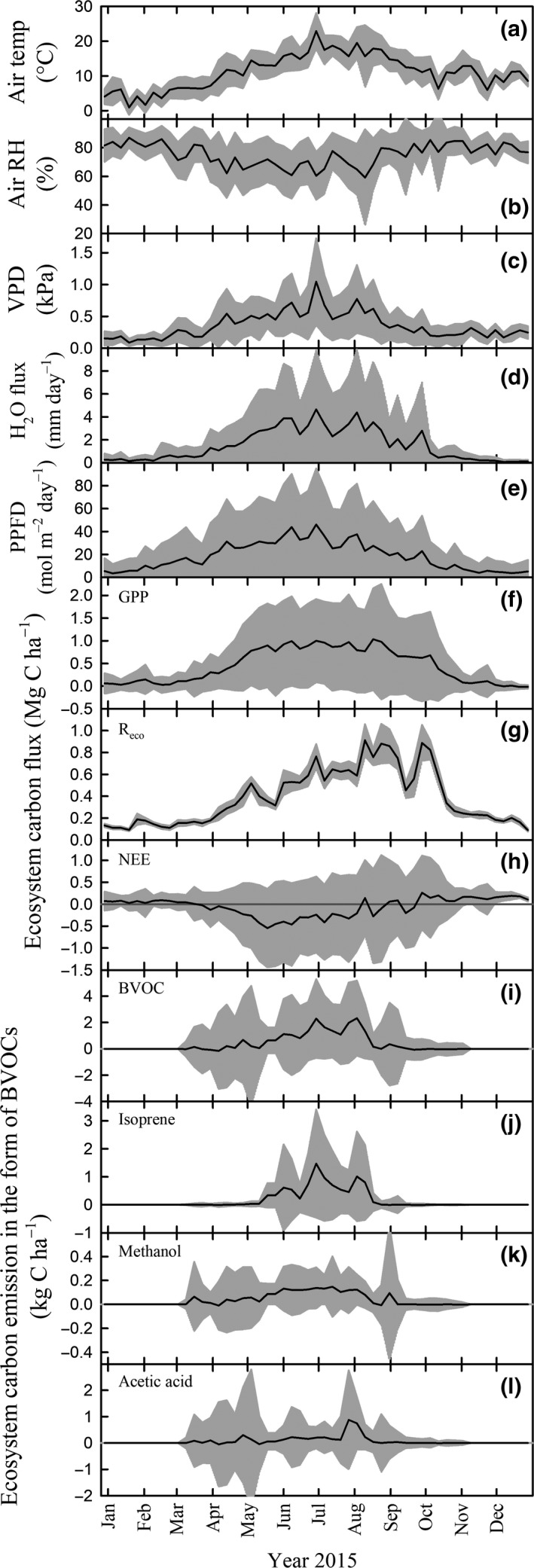
Weekly (a–c, e) meteorological data, (d) water evapotranspiration and (f–h) ecosystem carbon fluxes as CO_2_ and (i–l) as biogenic volatile organic compounds (BVOCs) during the year 2015. Values of the black line are the weekly average of 30‐min data and the gray area represents their standard deviation. Air temp, air temperature; RH, relative humidity; VPD, vapor pressure deficit; PPFD, photosynthetic photon flux density; R_eco_, ecosystem respiration; NEE, net ecosystem exchange; and GPP, gross primary production.

Carbon emissions in the form of BVOCs occurred during the growing season, reaching a maximum between June and mid‐August (Fig. 2i–l). This was the period of the year with weekly averaged highest mean air temperature (15.7–22.9 °C, Fig. 2a), lowest mean relative air humidity (60–77%, Fig. 2b), highest vapor pressure deficit (0.45–1.05 kPa, Fig. 2c), highest mean PPFD (33–46 mol m^−2^ day^−1^, Fig. 2e), and a high evapotranspiration (2.3–4.6 mm day^−1^, Fig. 2d).

Isoprene emission began few weeks after the onset of the growing season (Fig. 2j), denoting an increase in isoprene emissions as leaves developed until maximum leaf area index was achieved in mid‐August, before bud set (information retrieved from Vanbeveren *et al*. (2016)). Isoprene emission followed the same seasonal trend as VPD (Fig. 2c) and PPFD (Fig. 2e), which presented lower values in mid‐July than during the previous weeks. This lower emission was almost certainly due to a drop in photosynthetic activity during these days.

The highest carbon emissions as BVOCs peaked at 0.33 kg C ha^−1^ day^−1^ in weeks 27 (DOY 180–186, June 29–July 5) and 32 (DOY 215–221, August 3–9) (maxima in Fig. 2i) concurring with high air temperature, low relative humidity, high VPD, high H_2_O flux, high PPFD, and high GPP. These BVOC emission peaks were dominated by isoprene emissions (0.210 and 0.144 kg C ha^−1^ day^−1^, Fig. 2j), which represented 64% and 43% of the total BVOC carbon emission in weeks 27 and 32, respectively.

There was a heat wave from DOY 181–187 (June 30–July 6) characterized by at least five consecutive days with air temperature maxima over 25 °C, from which at least three days trespassed the 30 °C (as defined by the Royal Institute of Meteorology of Belgium; http://www.meteo.be/meteo). These extreme temperatures affected the isoprene emission by decreasing it when it was too hot, but not evapotranspiration nor CO_2_ assimilation. On DOY 181 (June 30), the highest isoprene emission of 0.376 kg C ha^−1^ (i.e., 6.275 mol ha^−1^) was observed (daytime air temperature of 25.2 ± 4.0 °C, maximum temperature of 27.8 °C, mean relative air humidity of 53%, mean VPD of 1.22 kPa, mean H_2_O flux of 5.6 mm day^−1^, and total PPFD of 52 mmol m^−2^). The following day, DOY 182 (July 1), was the hottest (daytime air temperature of 30.3 ± 4.6 °C and maximum of 34.5 °C) and driest (mean RH of 44% and mean VPD of 1.82 kPa) day of the year, along with 54 mmol m^−2^ of PPFD throughout the day. Although evapotranspiration was sustained (mean H_2_O flux of 6.3 mm day^−1^) by these not water‐limited poplars, the prolonged heat on DOY 182 (July 1) exceeded the threshold for optimal temperature. This led to a drop in photosynthetic activity and consequently a drop in isoprene emission to 0.255 kg C ha^−1^ (i.e., 4.247 mol ha^−1^).

Similar to isoprene, methanol was emitted throughout the growing season although the emission started earlier and lasted longer (DOY 75–233, March 16–August 21) than for isoprene. Overall, ecosystem carbon emitted as methanol was lower than as isoprene (Fig. 2k), but at a similar level in terms of molar quantities (Fig. 1). As for the seasonal dynamics, methanol emission increased during the beginning of the growing season and achieved a steady value during the summertime until the end of leaf growth in late August (Vanbeveren *et al*., 2016) similar to the seasonal dynamics of isoprene emissions. Carbon under the form of acetic acid was also emitted at a similar level than methanol and isoprene with an emission peak in the beginning of August (Fig. 2l).

### Diurnal patterns of isoprene emission, other BVOCs, and correlations with environmental parameters

**Figure 3:**
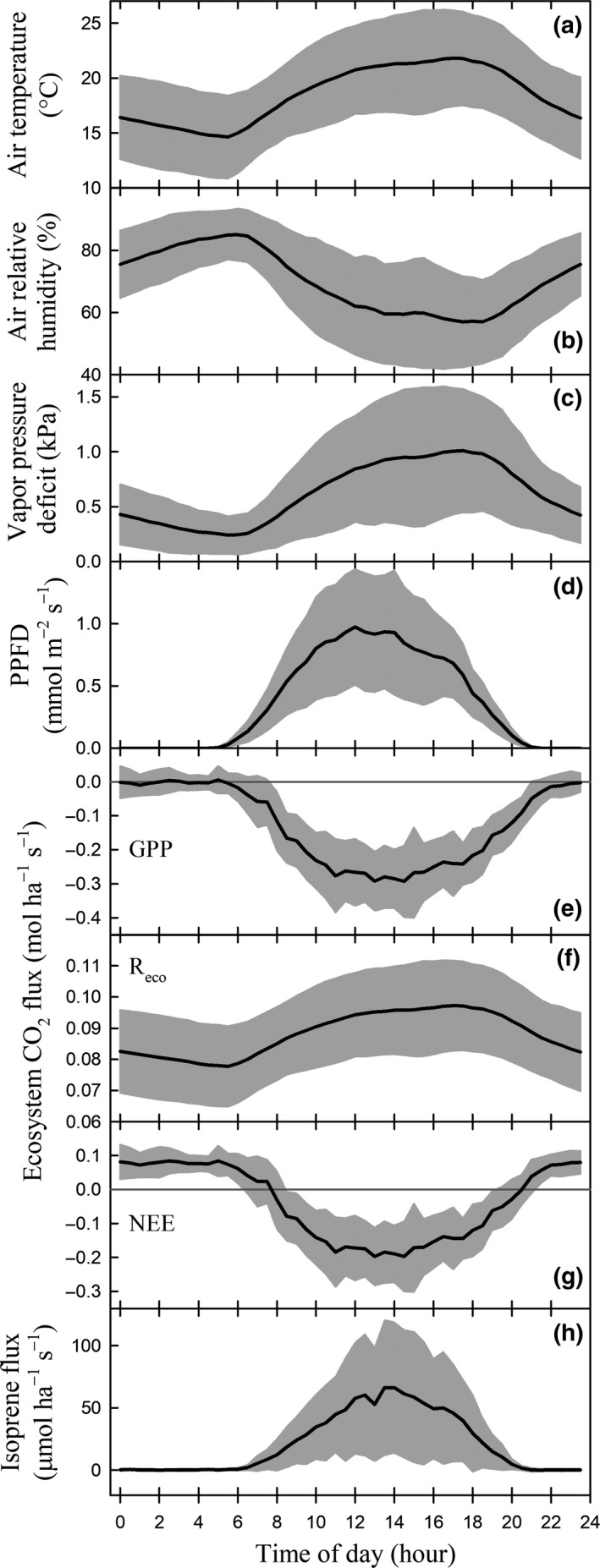
Diurnal pattern of (a–d) environmental parameters and molar fluxes of (e–g) CO_2_ and (h) isoprene in July 2015. The black lines represent the monthly averaged half‐hourly values and the shaded area covers the standard deviation among the 31 days at each half‐hour time window. Time is reported as local time = GMT+1, no summertime daylight saving time correction. In (c), vapor pressure deficit is a function of air temperature and air relative humidity. In (d), PPFD, photosynthetic photon flux density. In (e–g), GPP, gross primary production; R_eco_, ecosystem respiration; and NEE, net ecosystem exchange.

To compare the dynamics of BVOC emission rates with those of environmental variables, we selected the month of July, which was representative of the growing season. An average daily course has been graphically represented (Fig. 3). More data of the diurnal trends of 16 BVOCs, CO_2_ and H_2_O fluxes, and meteorological variables in other months (March–October) are found in Figs S3–S6.

**Figure 4:**
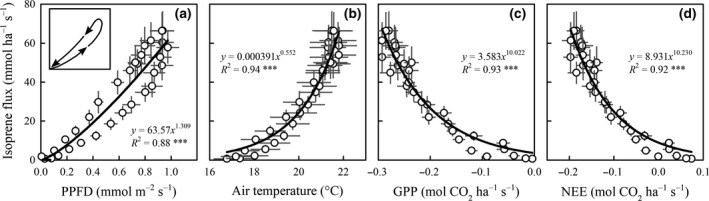
Correlations between isoprene emission and (a) PPFD (photosynthetic photon flux density), (b) air temperature, and (c, d) CO_2_ fluxes in July 2015. Dots are the monthly averaged half‐hourly values of each day; bars represent the standard error (*n* = 31 days) of each half‐hourly mean value. The data correspond to daytime (05:30 to 20:30), when isoprene flux was higher than zero. The inset in (a) emphasizes the hysteresis of the isoprene emission as a function of PPFD. GPP, gross primary production; and NEE, net ecosystem exchange. (***) denotes a significant correlation at the level of *P *<* *0.001.

A typical day in July began at 05:30 when PPFD lighted the top of the canopy; PPFD became maximum at about 12:00–14:00, and the day ended at 20:30 (Fig. 3d; local time = GMT+1, no summertime daylight saving time correction). Air temperature and VPD had their maxima (minimum for air RH) around 16:00 to 18:00 (Fig. 3a–c). The isoprene emission during the day started at 06:00, peaked around 13:00–14:00, and ended at 20:30 (Fig. 3h). We observed a consistent time lag of about 1:00 to 1:30 h in the isoprene emission maxima with respect to the PPFD maxima throughout the month of July. This resulted in a hysteretic correlation with PPFD (Fig. 4a).

The emission of other BVOCs presented a similar diurnal trend, especially pronounced during the months of June, July, and August. This clearly was the case for acetaldehyde, acetic acid, benzoic acid (Fig. S3), hexenal, formaldehyde, methyl ethyl ketone, methanol (Fig. S4), methyl vinyl ketone and methacrolein, pentenal, pentenone, and propene (Fig. S5). Other BVOCs presented less obvious diurnal trends. The month of October was in general low in BVOC emissions, and the month of April displayed high standard deviations over the monthly mean. The plantation was a net sink of formaldehyde (Fig. S4 h–n). The 30‐min flux data revealed a trend of a negative formaldehyde flux when its concentration in the air (VMR, volume mixing ratio) increased; the compensation point in the air was 0.53 ppb. As the measured formaldehyde VMR was always higher than the compensation point, the deposition was favored at the poplar plantation. Annual mean formaldehyde flux was −0.114 nmol m^−2^ s^−1^.

Isoprene emissions in July strongly correlated to air temperature and CO_2_ fluxes (Fig. 4b, d). GPP and NEE followed a similar diurnal trend as isoprene emissions and as PPFD, whereas the trend of R_eco_ followed the dynamics of air temperature and VPD (Fig. 3e–g). As expected, GPP correlated very well with PPFD, and R_eco_ with air temperature and VPD (Fig. S7). Similar trends for other months and BVOCs were observed, as shown in Figs S3–S6.

## Discussion

The proportion of carbon allocated to BVOC emissions highly depended on the temporal perspective as we observed from our one‐year‐long monitoring. The large variation in this proportion depending on the time period that is considered (week, month, year) clearly illustrated that the extrapolation of flux measurements over only a few weeks results in highly uncertain or wrong values. Several studies tried to quantify global BVOC emissions using modeling approaches (e.g., Ashworth *et al*. 2012, 2013b, Karl *et al*. 2009, Kesselmeier *et al*. 2002). Although these studies have some value, their simulated outcome and reliability are fairly questionable whether the data used for modeling and for extrapolation do not correctly capture the environmental conditions or whether they are based on results from short‐term monitoring (e.g., the extrapolation of short‐term leaf or branch enclosure studies under laboratory conditions to ecosystem‐scale BVOC fluxes and to annual carbon budgets). Bridging instantaneous leaf measurements to ecosystem‐scale fluxes remains therefore a major challenge because canopies are characterized by a high level of heterogeneity of temperature and PPFD absorption (Ashworth *et al*., 2013a).

As for the units of BVOC emissions, we consider it important to report values in both *mass of carbon* and *number of molecules*. Generally, atmospheric chemists and plant physiologists prefer to report fluxes in terms of number of molecules (e.g., μmol m^−2^ s^−1^). This allows to understand the chemical reactions within the leaf, the phyllosphere, or the atmosphere by their stoichiometry; emission rates can then be compared to the production of precursor metabolites, etc. On the other hand, scientists interested in biogeochemical cycles or in ecosystem productivity prefer to report these values based on mass of carbon, for which we have to count the number of carbon atoms in the structure of each volatile species. Two of the most emitted BVOCs in our ecosystem, isoprene and methanol, adequately illustrate the difference between both types of units. Reporting only the values in mass of carbon would give at first instance a biased view of the yield of these molecules in the atmosphere. Both contribute to subsequent reactions with hydroxyl radicals (^•^OH), although isoprene reacts much faster than methanol and also generates further products that can also react with ^•^OH.

### Percentage of assimilated carbon emitted as BVOCs

A previous study (Brilli *et al*., 2016) conducted in 2012 at the same plantation (first year of the second rotation) reported that carbon emitted as BVOCs was 0.8% of NEE during the growing season, a value slightly higher than our current results. The poplar plantation has evolved toward a stronger net sink of carbon throughout the three rotations (Zenone *et al*., 2015): The NEE values of the second year of each rotation were −95.6 g C m^−2^ in 2011, −275 g C m^−2^ in 2013, and −304 g C m^−2^ in 2015. The increasing carbon intake denotes that poplars could grow bigger at each rotation (achieving a higher leaf area index) helped by an already existing root system from the previous rotation. Despite this, the plantation was a net source of carbon on the first year of each rotation: NEE was 75.2 g C m^−2^ in 2010 (staring on DOY 152), 151 g C m^−2^ in 2012, and 70.2 g C m^−2^ in 2014 (value not yet published). This reflected a sustained R_eco_ carried out by a developed belowground tree biomass (Berhongaray *et al*., 2015) while GPP was suppressed due to the harvested aerial biomass, resulting in a carbon imbalance at the plantation. Accordingly, the percentage of carbon emitted as BVOCs may have also varied throughout the years and rotations. This makes it difficult to find a clear explanation to the variation in the carbon emitted as BVOCs between 2012 (the first year of the second rotation) and 2015 (the second year of the third rotation). The carbon emitted as BVOCs amounted to 0.49% of NEE during the same period studied by Brilli *et al*. (2016) in the year 2015, a smaller portion compared to the 0.8% calculated in 2012. As exposed previously, it is hard to accurately explain the effect of plantation age on the portion of carbon allocated to BVOCs in a recently established short‐rotation coppice plantation.

Other studies reported values higher than ours for the *Populus* genus: 0.5–2% of NEE was emitted as BVOCs in a hybrid poplar in Boulder (CO, USA) with *P*. *deltoides* × *P*. *trichocarpa* hybrids being stronger emitters than *P*. *deltoides* × *P*. *nigra* hybrids (Eller *et al*., 2012). Moreover, *P*. *deltoides* and *P*. *nigra* emitted 0.55–1.31% of NEE as isoprene at the leaf level under maximum photosynthetic capacity conditions in Rome, Italy (Guidolotti *et al*., 2011). In contrast, a mixed temperate forest (oak, red maple, red pine, and hemlock) in Petersham (MA, USA) released a 2% of annual NEE as isoprene emissions (Goldstein *et al*., 1998) and a high‐emitting nonstoring temperate oak forest in Ashland (MO, USA) emitted up to 5–10% of NEE as isoprene (Seco *et al*., 2015), while a coniferous *Pinus ponderosa* forest in the Sierra Nevada Mountains (CA, USA) emitted a 4% of the NEE annually as BVOC emissions (Bouvier‐Brown *et al*., 2012). The higher percentage in the latter case was most probably related to the higher amount of isoprenoids synthesized and stored in resin ducts in pine needles (Portillo‐Estrada *et al*., 2015a) that are absent in *Populus* leaves.

### Dynamics of isoprene emission and environmental drivers

Isoprene emission is light‐ and temperature‐dependent and related to ecosystem CO_2_ intake (i.e., GPP). However, the observed hysteresis of the isoprene emission as a function of PPFD indicated a small lag in the response of the emission to the light input; this was also evident in the relationships of GPP with PPFD, and of R_eco_ with VPD. This physiological hysteresis is due to a delay in the stomatal conductance response to light stimuli, already reported for *Populus* (Ceulemans *et al*., 1988). This lag in response could also partially reflect the time between leaf‐level emissions and the detection by the eddy covariance tower due to developed mechanical turbulence and – more likely– the gas diffusion through the canopy.

From the long‐term perspective, isoprene emissions did not represent a major carbon loss from this SRC plantation although the emission can be significant on a shorter time scale during hot days with optimal conditions for high photosynthetic activity (e.g., DOY 181) (Goldstein *et al*., 1998). The decrease in isoprene emission at DOY 182 (July 1) could reflect the temperature dependency of isoprene emission and its decay when leaf temperature trespasses the optimal temperature for isoprene synthase and DMADP pool size, as reported for hybrid poplar (Rasulov *et al*., 2010). However, the productivity of the poplars at the SRC plantation was not substantially affected by the heat wave. This resulted in a sustained evapotranspiration of the poplars during the period of the lowest water table level (Horemans *et al*., 2017).

### Biological controls of the emission of other BVOCs

The presence of methanol in the BVOC emission blend of poplar leaves is known (Hüve *et al*., 2007). Methanol emission is related to cell elongation and division, and occurs more intensively in younger and expanding leaves (Brilli *et al*., 2011; Portillo‐Estrada *et al*., 2017), remarkably in early spring, even before isoprene is emitted (Ghirardo *et al*., 2011). Therefore, a plantation of poplars, a fast‐growing species that quickly unfolds its leaves in spring, generates a burst of methanol emission due to the many young and expanding leaves during this period. We observed that methanol – after spring – was steadily emitted during the growing season due to the acropetal development of poplar shoots. The latter implies that the leaf cohort always had a share of young and expanding leaves during the growing season. Later in the growing season, methanol emission could represent other leaf degenerative processes as leaf wounding by herbivores (Portillo‐Estrada *et al*., 2015b), rust infection (Jiang *et al*., 2016), or programmed leaf senescence (Mozaffar *et al*., 2017).

Some of the BVOCs detected are oxygenated volatiles that are often by‐products of key metabolic processes (Niinemets *et al*., 2014). This is the case of formic and acetic acid, acetone, formaldehyde, acetaldehyde, methanol, and ethanol (Seco *et al*., 2007). Their diurnal emission trends reflected a synchrony with the diurnal trends of the meteorological variables and plant metabolism, measured as CO_2_ fixation and emission of isoprene. On the other hand, the diurnal trends of methyl ethyl ketone and methyl vinyl ketone + methacrolein reflected the oxidation of isoprene in the lower atmosphere or within the leaves (Brilli *et al*., 2016).

Ethanol is produced in anoxic conditions as a fermentation product of pyruvate (e.g., bacteria in the soil and plant roots). When produced in plant roots, it is transported in the xylem thanks to the transpiration flow and eventually emitted. Ethanol also oxidizes within the leaves to acetaldehyde and then to acetic acid (Kreuzwieser *et al*., 1999; Kreuzwieser & Rennenberg, 2013). Plant emission of acetic acid is light‐dependent (Kesselmeier & Staudt, 1999). We did, however, not find any significant relationship between net daily emissions of acetic acid and daily cumulated PPFD, despite the clear diurnal trends in June, July, and August. As there was no robust correlation with acetaldehyde, we assumed that acetic acid emission was more related to air and soil temperatures than to incoming light; it is mostly caused by soil microbial activity and litter decomposition (Gray *et al*., 2010; Hafner *et al*., 2013), because leaf litter is left to decompose on the ground of the plantation.

The poplar plantation was a net sink of formaldehyde. We found a higher average deposition (−0.055 nmol m^−2^ s^−1^, *n* = 7200) than in a previous study on the same SRC plantation (Brilli *et al*. (2016); −0.015 nmol m^−2^ s^−1^) for the period of June‐October. This might probably be explained by differences in the developmental status of the soil structure, more developed root systems over time, and the accumulated increase in soil carbon content over the years observed at the site since its establishment (Berhongaray *et al*., 2017). More interesting is that the average deposition increased to −0.114 nmol m^−2^ s^−1^ when accounting over the whole year, because formaldehyde was deposited also in April and May. This confirms that monitoring BVOC emissions before the start of the growing season is important. Formaldehyde, known to be a human carcinogen, does not accumulate in the atmosphere. Either it reacts fast with oxygen to form formic acid – which has a lower toxicity – or it is broken down by sunlight or by soil bacteria, or it is deposited to the vegetation facilitated by stomatal conductance and by a high volume mixing ratio in the air (Brilli *et al*., 2016).

This study confirmed the low significance of BVOC emissions in the carbon cycle compared to NEE; there is, thus, not much to correct the net ecosystem carbon exchange for BVOC emissions. However, our study emphasizes the importance of tracing BVOC emissions, their compounds, and amounts, to quantify the reactive carbon emitted to the atmosphere. Isoprene and methanol are the dominant molecules in the annual BVOC emissions, and the isoprene emissions are closely related to PPFD and to CO_2_ fluxes.

## Supporting information


**Figure S1.** Scheme of volatile organic compound data acquisition with the PTR‐TOF‐MS.
**Figure S2.** Example of analysis of a multipeak with the ‘Multipeak’ built‐in tool in the software PTR‐MS Viewer v3.2 (Ionicon, Innsbruck, Austria).
**Figure S3.** Diurnal trends per month of BVOC emissions at the poplar short‐rotation plantation in year 2015. Average ± SE (*n* = days of month).
**Figure S4.** Diurnal trends per month of BVOC emissions at the poplar short‐rotation plantation in year 2015. Average ± SE (*n* = days of month).
**Figure S5.** Diurnal trends per month of BVOC emissions at the poplar short‐rotation plantation in year 2015. Average ± SE (*n* = days of month).
**Figure S6.** Diurnal trends per month of environmental parameters, water and CO_2_ fluxes as GPP (gross primary production), R_eco_ (ecosystem respiration), and NEE (net ecosystem exchange).
**Figure S7.** Correlations between CO_2_ fluxes and environmental parameters in July 2015.
**Table S1.** List of ion masses and molecular formulae measured with the PTR‐TOF‐MS.
