# Supplementary material for: Contribution of volatile organic compound fluxes to the ecosystem carbon budget of a poplar short‐rotation plantation
Source: Glob Change Biol Bioenergy. 2018 Mar 25;10(6):405–14. doi: 10.1111/gcbb.12506 (PMC5993229; doi:10.1111/gcbb.12506)
Supplement: Supplementary file 1 — Figure S1. Scheme of volatile organic compound data acquisition with the PTR‐TOF‐MS. Figure S2. Example of analysis of a multipeak with the ‘Multipeak’ built‐in tool in the software PTR‐MS Viewer v3.2 (Ionicon, Innsbruck, Austria). Figure S3. Diurnal trends per month of BVOC emissions at the poplar short‐rotation plantation in year 2015. Average ± SE (n = days of month). Figure S4. Diurnal trends per month of BVOC emissions at the poplar short‐rotation plantation in year 2015. Average ± SE (n = days of month). Figure S5. Diurnal trends per month of BVOC emissions at the poplar short‐rotation plantation in year 2015. Average ± SE (n = days of month). Figure S6. Diurnal trends per month of environmental parameters, water and CO2 fluxes as GPP (gross primary production), Reco (ecosystem respiration), and NEE (net ecosystem exchange). Figure S7. Correlations between CO2 fluxes and environmental parameters in July 2015. Table S1. List of ion masses and molecular formulae measured with the PTR‐TOF‐MS. [file GCBB-10-405-s001.docx]

# **Supplementary material**

**Table S1**. List of ion masses and molecular formulae measured with the PTR-TOF-MS. The fragmentation rates of each molecule at a given field density ratio (E/N) were used to calculate the reaction rates of the parent ions. The most probable molecule structure, thus compound name, in the first column was retrieved from the direct calibration, from GC-MS analyses conducted at the study site in 2012 or from the literature on poplar leaf emissions and similar eddy covariance studies. The natural abundance (%) of each protonated molecule containing ^1^H, ^12^C, ^14^N, and ^16^O can be used to correct the BVOC fluxes and take into account the amounts of molecules containing isotopes ^2^H, ^13^C, ^15^N, ^17^O, and ^18^O.

| Most probable compound structure | Molecular formula of the protonated compound | Peak centre (protonated molecular mass) | Abundance ratio (%) |
| --- | --- | --- | --- |
| Formaldehyde | (CH_2_O)H^+^ | 31.0178 | 98.6639 |
| Methanol | (CH_4_O)H^+^ | 33.0335 | 98.6414 |
| Acetonitrile | (C_2_H_3_N)H^+^ | 42.0338 | 97.4866 |
| Propene | (C_3_H_6_)H^+^ | 43.0542 | 96.7703 |
| Acetaldehyde | (C_2_H_4_O)H^+^ | 45.0335 | 97.5938 |
| Formic acid | (CH_2_O_2_)H^+^ | 47.0128 | 98.4243 |
| Ethanol | (C_2_H_6_O)H^+^ | 47.0491 | 97.5715 |
| Acetone | (C_3_H_6_O)H^+^ | 59.0491 | 96.5353 |
| Acetic acid (+ fragment) | (C_2_H_4_O_2_)H^+^  (C_2_H_2_O)H^+^ | 61.0284  43.0178 | 97.3568  97.6161 |
| Isoprene (+ fragment) | (C_5_H_8_)H^+^  (C_3_H_4_)H^+^ | 69.0699  41.0386 | 94.7041  96.7924 |
| Methyl vinyl ketone + methacrolein | (C_4_H_6_O)H^+^ | 71.0491 | 95.5101 |
| Methyl ethyl ketone | (C_4_H_8_O)H^+^ | 73.0648 | 95.4882 |
| Benzene | (C_6_H_6_)H^+^ | 79.0542 | 93.7198 |
| Pentenone | (C_5_H_8_O)H^+^ | 85.0648 | 94.4741 |
| Pentenol | (C_5_H_10_O)H^+^ | 87.0804 | 94.4525 |
| Pentanol (+ fragment) | (C_5_H_12_O)H^+^  (C_5_H_10_)H^+^ | 89.0961  71.0855 | 94.4310  94.6825 |
| Toluene + cymene | (C_7_H_8_)H^+^ | 93.0699 | 92.7033 |
| Furfural | (C_5_H_4_O_2_)H^+^ | 97.0290 | 94.2878 |
| Hexenal (+ fragments) | (C_6_H_10_O)H^+^  (C_6_H_8_)H^+^  (C_3_H_4_O)H^+^ | 99.0804  81.0699  57.0335 | 93.4495  93.6984  96.5573 |
| Hexenol + hexanal (+ fragment) | (C_6_H_12_O)H^+^  (C_6_H_10_)H^+^ | 101.0961  83.0855 | 93.4281  93.6769 |
| Hexanol (+ fragment) | (C_6_H_14_O)H^+^  (C_6_H_12_)H^+^ | 103.1117  85.1012 | 93.4067  93.6555 |
| Styrene | (C_8_H_8_)H^+^ | 105.0699 | 91.7188 |
| Benzaldehyde | (C_7_H_6_O)H^+^ | 107.0491 | 92.4993 |
| Xylene | (C_8_H_10_)H^+^ | 107.0855 | 91.6978 |
| Benzoic acid | (C_7_H_6_O_2_)H^+^ | 123.0441 | 92.2747 |
| Octenal | (C_8_H_14_O)H^+^ | 127.1117 | 91.4333 |
| Monoterpenes (+ fragments) | (C_10_H_16_)H^+^  (C_7_H_10_)H^+^  (C_6_H_8_)H^+^ | 137.1325  95.0855  81.0699 | 89.6990  92.6821  93.6984 |
| Hexyl acetate | (C_8_H_16_O_2_)H^+^ | 145.1223 | 91.1904 |
| Dimethyl nonatriene (DMNT) | (C_11_H_18_)H^+^ | 151.1481 | 88.7261 |
| Methyl salicylate | (C_8_H_8_O_3_)H^+^ | 153.0546 | 91.0522 |
| Monoterpene alcohol | (C_10_H_18_O)H^+^ | 155.1430 | 89.4607 |
| Ethyl salicylate | (C_9_H_10_O_3_)H^+^ | 167.0703 | 90.0646 |
| Sesquiterpenes (+ fragment) | (C_15_H_24_)H^+^  (C_8_H_14_)H^+^ | 205.1951  111.1168 | 84.9583  91.6559 |
| Jasmonic acid | (C_12_H_18_O_3_)H^+^ | 211.1329 | 87.1458 |
| Ionol | (C_15_H_24_O)H^+^ | 221.1900 | 84.7520 |
| Methyl jasmonate | (C_13_H_20_O_3_)H^+^ | 225.1485 | 86.2006 |


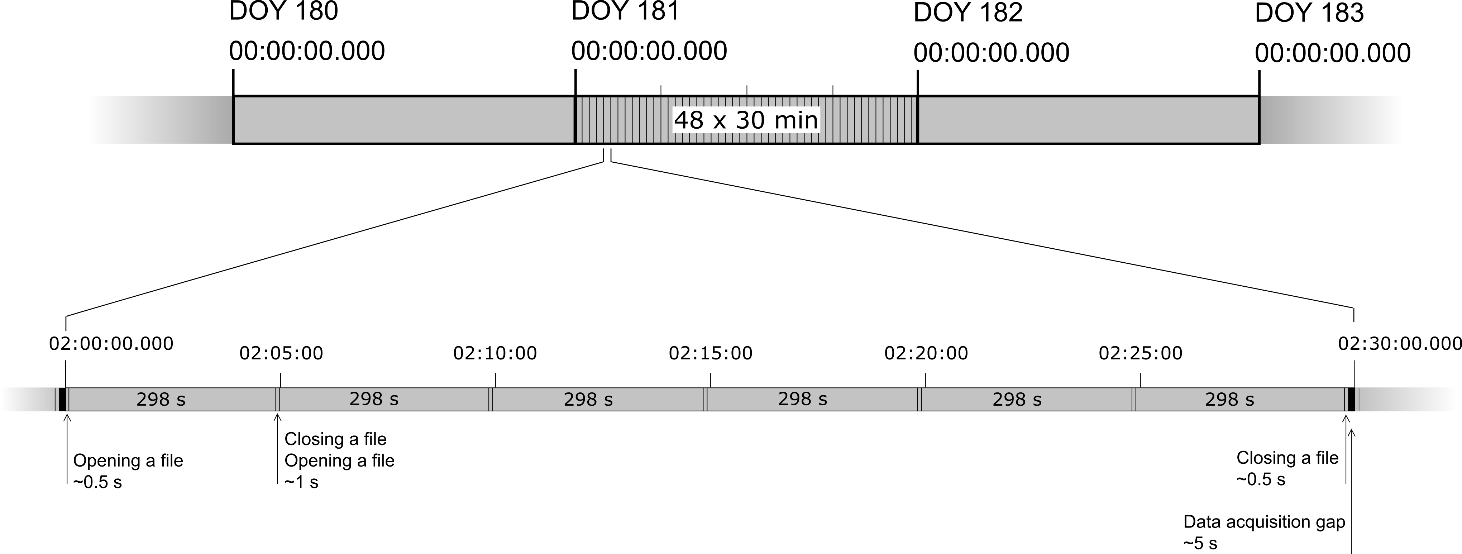


**Figure S1**. Scheme of volatile organic compound data acquisition with the PTR-TOF-MS. Detailed timing during a 30-min step of recording files, data loss during closing and opening files, and the data acquisition gap added at the end of the 30 min to match the data acquisition of the following 30 min step to the computer clock. The data were recorded in six subsequent 298 s files each half hour. Due to the large size of the data files (50-60 Mb), closing and starting a new data file generated a data gap of ~1 s. At the end of each half hour, a recording gap of ~5 s was allowed to adjust for these delays, so the next file corresponding to the following half hour would start matching the clock at HH:00 or HH:30, as set up in the automated recording algorithm.

**
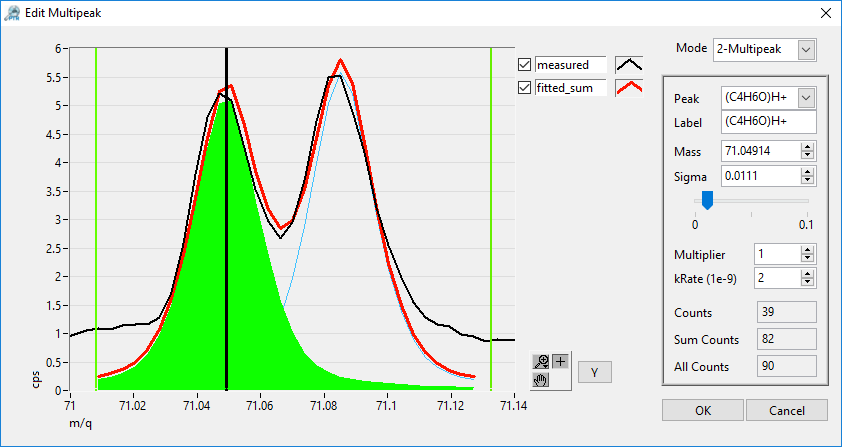
**

**Figure S2**. Example of analysis of a multipeak with the “Multipeak” built-in tool in the software PTR-MS Viewer v3.2 (Ionicon, Innsbruck, Austria). In this example, ions (C_4_H_6_O)H^+^ and (C_5_H_10_)H^+^ with adjacent peak centres at *m*/*z* 71.0491 and 71.0855, have overlapping peak tails. The tool allows to match the signal of both ions (in green and blue) to the measured signal (in counts per second) in an average spectrum (black line). The sigma of the Gaussian curve is tuned to match the sum of the signals of both ions (in red) to the measured data. These fitting parameters are used afterwards to calculate the concentrations of both ions independently.


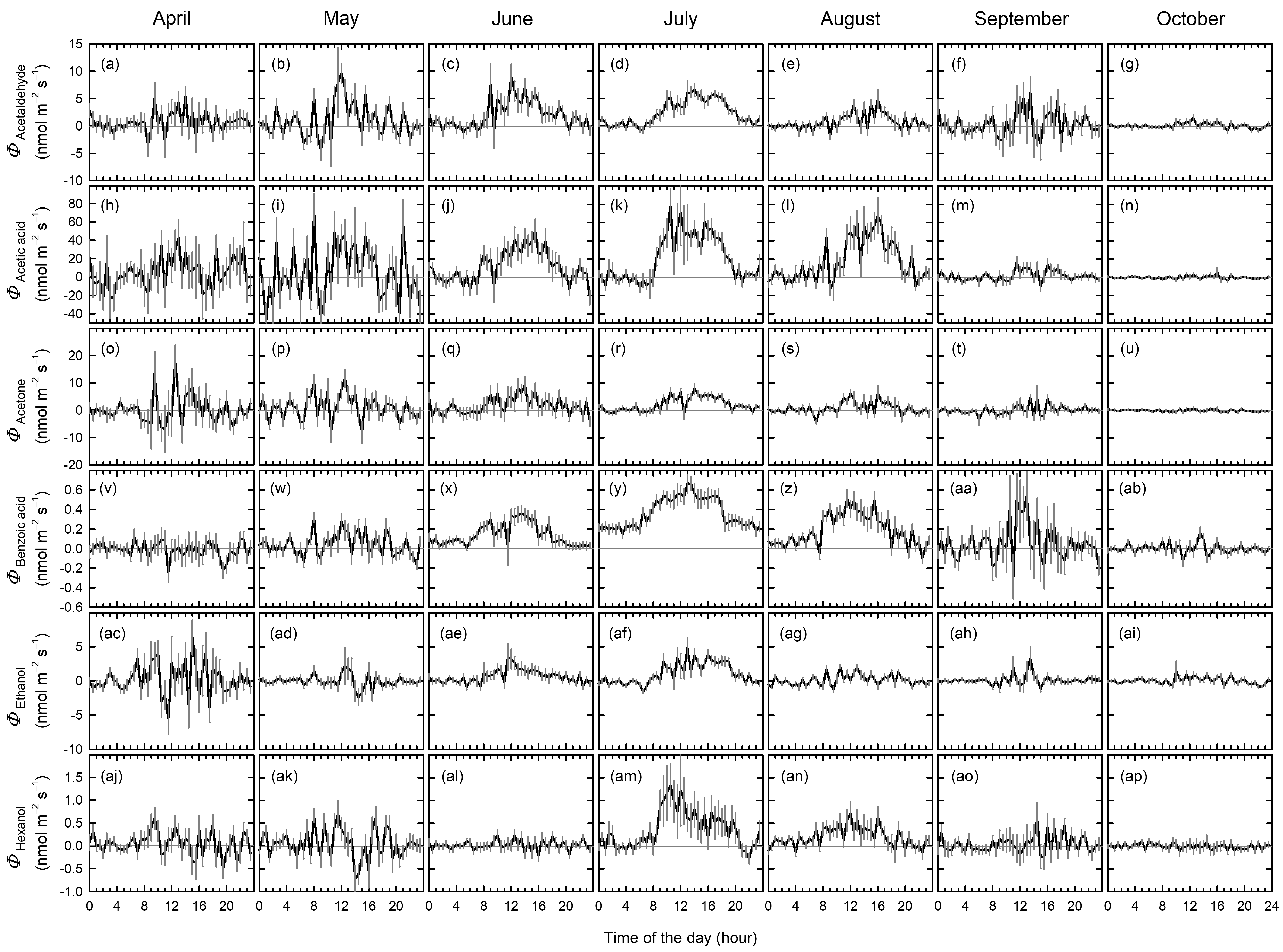


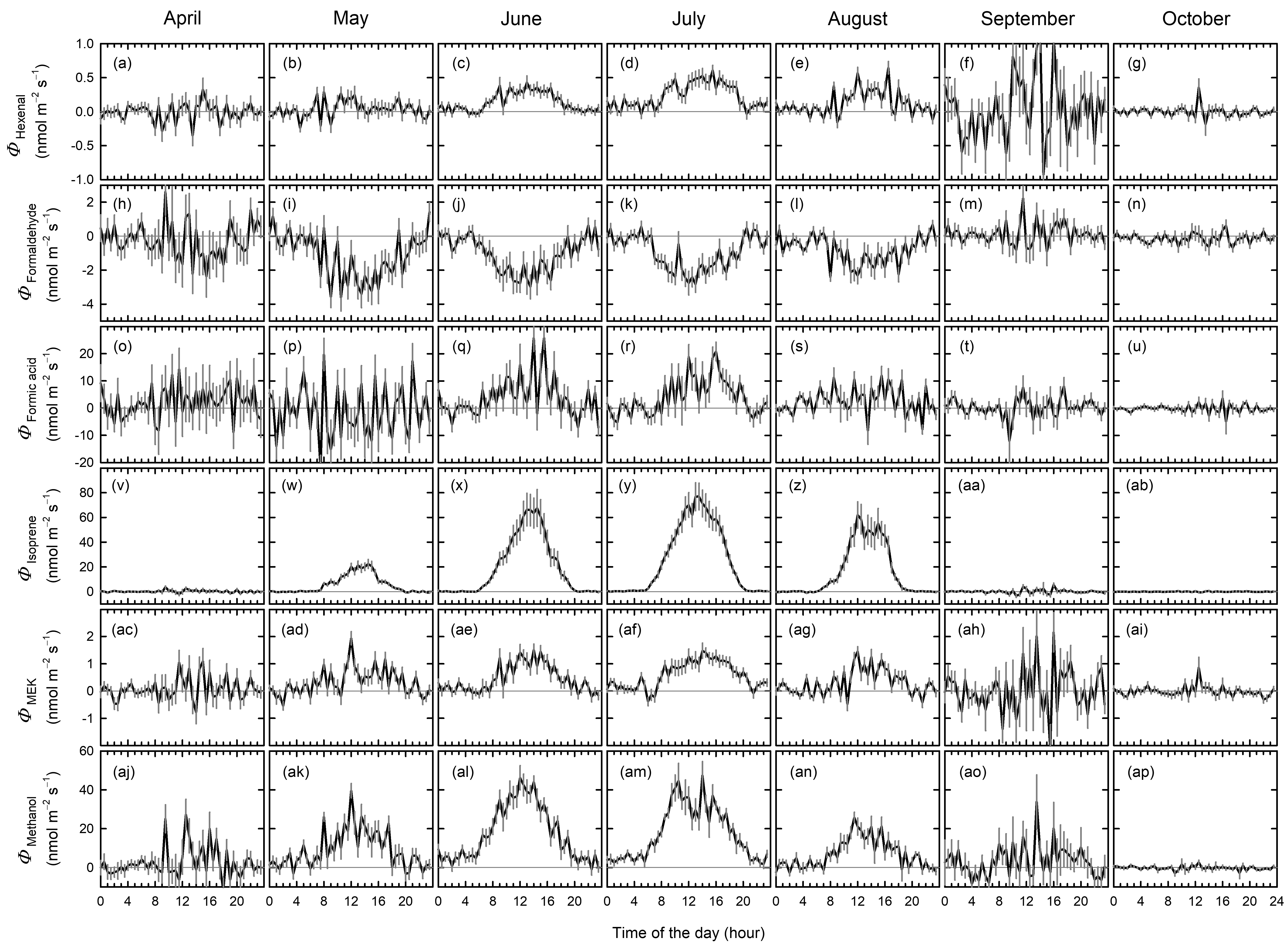


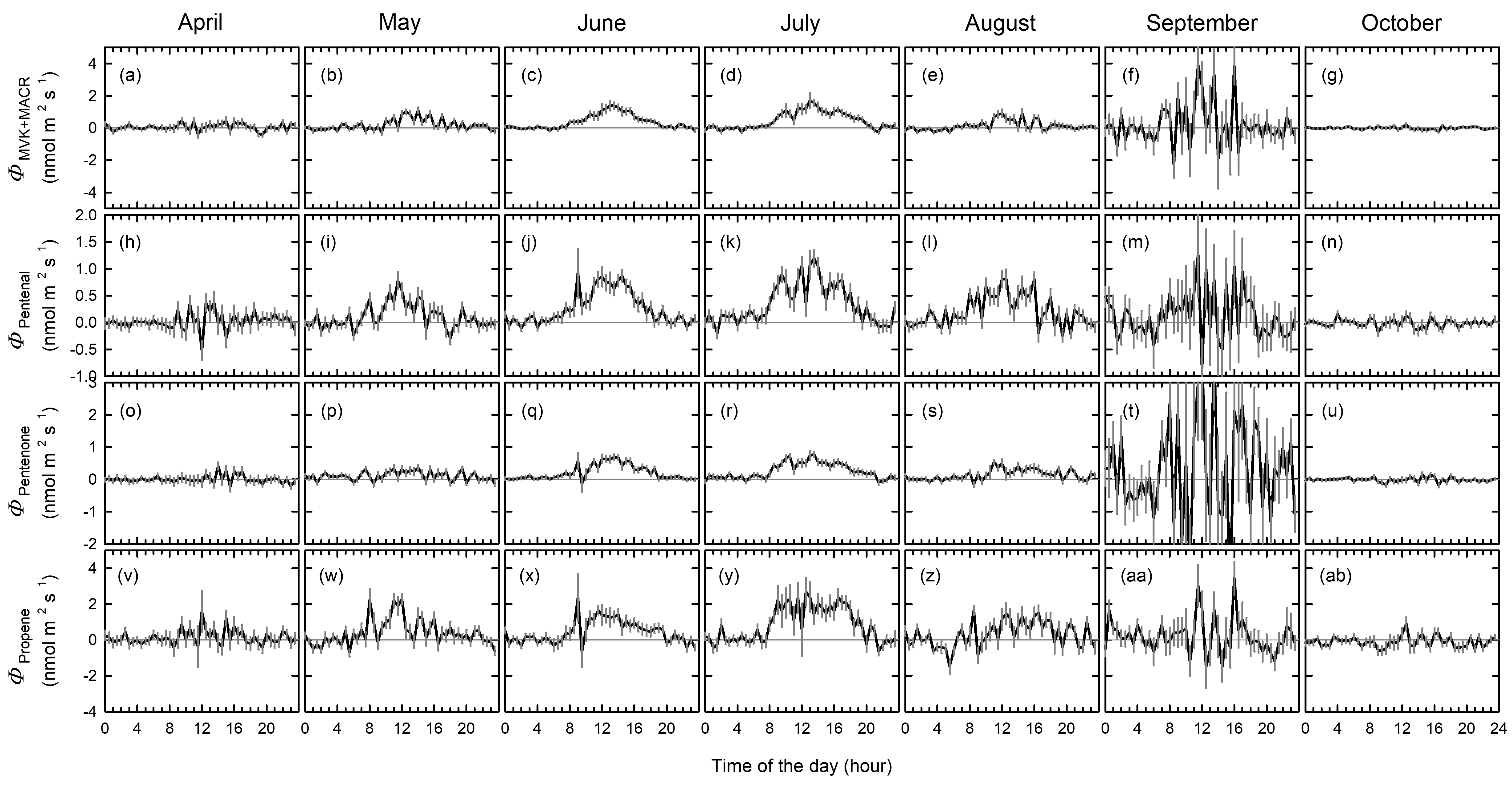


**Figure S3**. Diurnal trends per month of BVOC emissions at the poplar short-rotation plantation in year 2015. Average ± SE (n = days of month).

**Figure S4**. Diurnal trends per month of BVOC emissions at the poplar short-rotation plantation in year 2015. Average ± SE (n = days of month).

**Figure S5**. Diurnal trends per month of BVOC emissions at the poplar short-rotation plantation in year 2015. Average ± SE (n = days of month).


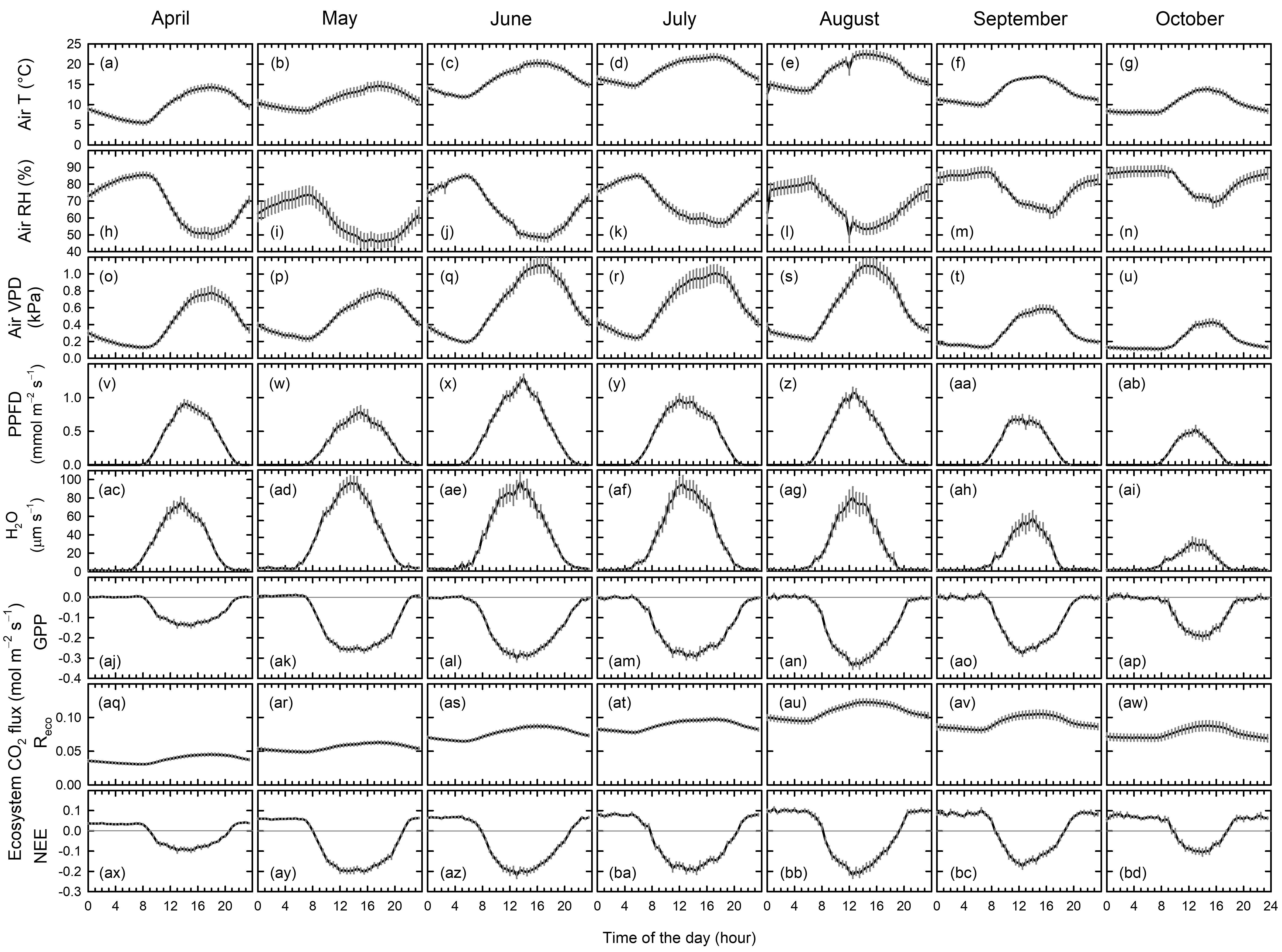


**Figure S6**. Diurnal trends per month of environmental parameters, water and CO_2_ fluxes as GPP (gross primary production), R_eco_ (ecosystem respiration), and NEE (net ecosystem exchange). Average ± SE (n = days of month).


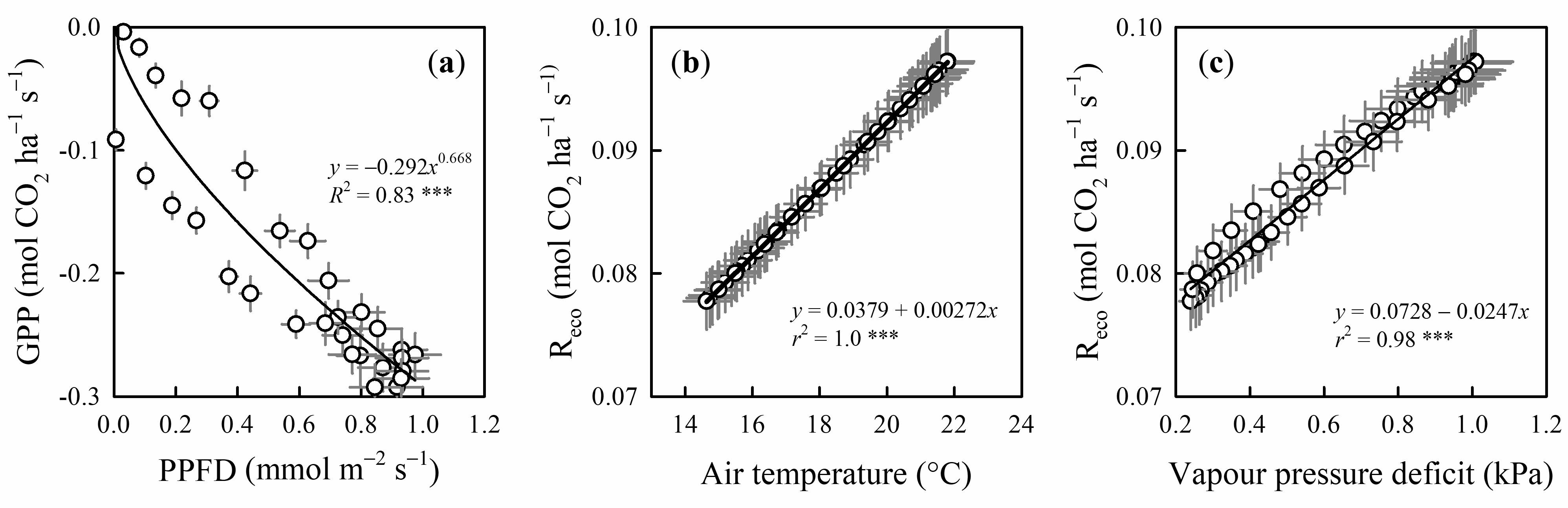


**Figure S7**. Correlations between CO_2_ fluxes and environmental parameters in July 2015. Dots are the monthly-averaged half-hourly values of each day; bars represent the standard error (n = 31 days) of each half-hourly mean value. In (a) the data correspond to daytime (05:30 to 20:30), when PPFD was higher than zero. GPP = gross primary production, R_eco_ = ecosystem respiration. (***) denotes a correlation significance level of *p* < 0.001.
